# Commentary on ‘Metabolic reprogramming‐associated genes predict overall survival for rectal cancer’

**DOI:** 10.1111/jcmm.15938

**Published:** 2020-10-22

**Authors:** Ruihuan Shen

**Affiliations:** ^1^ Department of Pediatric Intensive Care Unit National Center for Cardiovascular Disease and Fuwai Hospital Chinese Academy of Medical Sciences Peking Union Medical College Beijing China

**Keywords:** hypothesis, statistics, type I error

## Abstract

The current paper is a commentary on the Metabolic reprogramming‐associated genes predict overall survival for rectal cancer (Jian‐Qing Lin et al 2020). The authors concluded that ‘Patients with high‐risk demonstrated significantly poorer survival outcomes than patients with low‐risk in the TCGA database. Also, patients with high‐risk still showed significantly poorer survival outcomes than patients with low‐risk in the GEO database’. But the figure 3 in their published paper, ‘Survival analyses for the prognostic metabolic genes in rectal cancer’, presented that there was type I error in their study during the hypothesis testing process, obviously.

Hypothesis testing was the process of using sample data to test hypotheses. The purpose of this test was to present evidence that this hypothesis was supported by the data being tested. The null hypothesis (H0) was that there is no statistical significance between the two populations or data sets considered in the hypothesis.^1^ In general, however, researchers would like to attempt to disprove the H0.

In an ideal world, the H0 should never be rejected if it is considered to be true, and it should always be rejected if it is considered to be false. However, errors can occur in some cases.

A type I error was a precise technical term used in statistics to describe particular flaws during the hypothesis testing process, in which the wrong decision that was made when a test rejects a accurate H0.^1^ Type I error can be deemed as the error of excessive scepticism. And it was usually numeric equivalent to the significance level of a test.

In the study of Jian‐Qing Lin et al,^2^ the *P*‐value showed in the figure 3A and figure 3B was equal to 9.738e‐05 and 2.143e‐01, respectively. Obviously, the H0 should not be rejected. As a consequence, type I error occurs, so that the conclusion based on this hypothesis testing process may be not valid.

Then, our task group had tried to repeat the data analysis procedure. However, the opposite conclusions were drawn from our study (the data have not yet been published). These differences can be attributed mainly to the misunderstanding of the type I error we considered above. Furthermore, the method of Validation of TCGA survival analysis by utilizing data from the GEO database presented by author was too simple to repeat the data analysis procedure according to the method recommended by author, and the significance level of the study was not stated clearly in advance to define how the small the *P*‐value must be in order to reject the H0, resulting in our opinions were divided on the matter.

## CONFLICT OF INTEREST

The author states that there are no competing interests.

## AUTHOR CONTRIBUTION

This article was independently completed by Ruihuan Shen.

## Data Availability

The current paper is a commentary, so our paper is exempt from Data sharing.
